# Modulation of the executive control network by anodal tDCS over the left dorsolateral prefrontal cortex improves task shielding in dual tasking

**DOI:** 10.1038/s41598-023-33057-7

**Published:** 2023-04-15

**Authors:** Devu Mahesan, Daria Antonenko, Agnes Flöel, Rico Fischer

**Affiliations:** 1 Department of Psychology, University of Greifswald, Franz-Mehring-Strasse 47, 17489 Greifswald, Germany; 2 Department of Neurology, University Medicine Greifswald, Greifswald, Germany; 3 German Centre for Neurodegenerative Diseases (DZNE) Standort Greifswald, Greifswald, Germany

**Keywords:** Cognitive control, Human behaviour

## Abstract

Task shielding is an important executive control demand in dual-task performance enabling the segregation of stimulus–response translation processes in each task to minimize between-task interference. Although neuroimaging studies have shown activity in left dorsolateral prefrontal cortex (dlPFC) during various multitasking performances, the specific role of dlPFC in task shielding, and whether non-invasive brain stimulation (NIBS) may facilitate task shielding remains unclear. We therefore applied a single-blind, crossover sham-controlled design in which 34 participants performed a dual-task experiment with either anodal transcranial direct current stimulation (atDCS, 1 mA, 20 min) or sham tDCS (1 mA, 30 s) over left dlPFC. Task shielding was assessed by the backward-crosstalk effect, indicating the extent of between-task interference in dual tasks. Between-task interference was largest at high temporal overlap between tasks, i.e., at short stimulus onset asynchrony (SOA). Most importantly, in these conditions of highest multitasking demands, atDCS compared to sham stimulation significantly reduced between-task interference in error rates. These findings extend previous neuroimaging evidence and support modulation of successful task shielding through a conventional tDCS setup with anodal electrode over the left dlPFC. Moreover, our results demonstrate that NIBS can improve shielding of the prioritized task processing, especially in conditions of highest vulnerability to between-task interference.

## Introduction

Multitasking is required in a variety of contexts in modern life, ranging from multimedia use to vehicle operation and scheduling of complex task sequences in an emergency room. Yet, performing even two simple cognitive tasks at the same time often results in between-task interference, e.g., when the engagement in an additional task impacts the processing of a prioritized task. Executive control processes, subsumed under the term task shielding serve to reduce this between-task interference by ensuring the correct binding of task-specific stimulus codes to respective response codes in each task to minimize the risk of response reversals and/or confusion^1^. Because successful multitasking performance relies on a number of executive control functions, such as maintenance, monitoring, inter-task coordination, task segregation, or resource allocation^1–5^, attempts to improve multitasking abilities specifically target the optimization of the respective executive control components^6–8^.

Recent functional neuroimaging studies confirm a close link between dual-task performance and neural activity in brain regions associated with executive control, such as the dorsolateral prefrontal cortex (dlPFC, e.g.,^9–15^). Stelzel et al.^16^, for example, reported enhanced functional connectivity between the dlPFC and sensory regions relevant for Task 1 processing when both tasks shared high temporal overlap, suggesting increased task shielding in conditions of highest multitasking demands. Furthermore, neural correlates of training-induced improvements in dual-tasking have been attributed to higher grey matter volumes^17^, increased efficiency in neural information processing^6^, or increased separation of task representations in the frontoparietal-subcortical networks, including the left dlPFC as a central network hub^18^.

Non-invasive brain stimulation techniques (NIBS), such as transcranial direct current stimulation (tDCS), have been applied to improve dual-task performance (e.g.,^19–23^). tDCS is a NIBS technique that applies a constant, low-amplitude electrical current using two or more electrodes on the scalp. Depending on the polarity of the current, the neural firings can either increase or decrease cortical excitability. In most cases, the beneficial effects on cognitive functions are reported for anodal tDCS (atDCS), which increases cortical excitability through depolarization^24,25^. The application of atDCS has revealed behavioral modification and improved cognitive functions such as working memory^26,27^ and attention^28,29^ in healthy (see^30^ for a review) and neurological patients (see^31^ for a review) and thus offers a promising tool to improve task shielding and to reduce between-task interference in dual-task performance.

Studies focusing on improving executive control in dual tasks with tDCS have supported the claim that the dlPFC plays a central role in dual-task performance, especially when high demands on executive functioning are required (e.g.,^22,23^). Most studies, however, have focused on comparing dual-task to single-task performance to assess dual-task costs^19–21,32^ or have addressed different executive control functions, such as task order control in dual tasks^22,23^. Although their findings generally demonstrate that NIBS over the left lPFC can facilitate dual-task performance, the applied dual-task paradigms, stimulation type, and protocols, as well as the investigated control functions differed greatly between these studies. It, therefore, remains unclear whether applying tDCS over left dlPFC can improve the executive control function of task shielding and thus reduce between-task interference in dual-task performance.

In the present study, we aimed to test whether the application of atDCS over the left dlPFC can improve task shielding. We used a dual-task paradigm, in which both tasks were presented in close temporal proximity and shared processing similarities to give rise to crosstalk between the two tasks. Effective task shielding can be inferred from the size of the so-called backward crosstalk effect (BCE) that reflects to which extent Task 1 processing is affected by simultaneous Task 2 processing. Importantly, the smaller this backward crosstalk onto Task 1 performance, the more effective is task shielding and interference control^33–37^. In two different experimental sessions, participants received either atDCS or sham tDCS over left dlPFC. As atDCS increases cortical excitability^24,25^, we hypothesized that atDCS (as compared to sham tDCS) during dual-task performance would modulate the underlying executive control network resulting in a reduced BCE, indicating improved task shielding.

## Methods

### Participants

Forty-one participants signed up for participation in the experiment. Data sets of seven participants were not usable for analyses because of technical problems during recording (one participant), not completing both sessions (four participants), difficulties following task instructions, and obvious signs of disinterest in participation (two participants). The final sample consisted of thirty-four healthy participants (27 females; mean age: 22.4 years [*SD* = 2.2 years, *range* = 19–28 years]). After completion of both sessions, participants received monetary compensation of €20 or course credits. The study was conducted according to the Declaration of Helsinki and was approved by the local Ethics Committee at the University Medicine Greifswald (BB 144/18). Participants had normal or corrected-to-normal vision and gave informed, written consent before data collection. Via pre-screening, participants reported no history of medical, neurological, or psychiatric diseases, no sleep disorders, alcohol or substance abuse, and no treatment with medication acting primarily on the central nervous system.

### Study overview

The study was conducted as a single-blind, crossover, sham-controlled trial. Each participant completed two sessions on separate days, with an average of 7 days between the two sessions. They received either atDCS or sham tDCS with the order of stimulation conditions being counterbalanced, i.e., half of the participants received atDCS, whereas the other half received sham tDCS for their first session. Each session lasted for about 60 min, including the tDCS setup.

### Materials and methods

#### Behavioral task

The dual task consisted of a visual discrimination task (Task 1) and an auditory discrimination task (Task 2), which were performed in close succession. In order to increase processing similarities between the two tasks as the basis for crosstalk^38^, a correspondence between the stimuli of both tasks was implemented. Participants responded to the identity of the letter (H or T) in Task 1 and the frequency of a tone (high or low) in Task 2. The high tone corresponded to the letter H, which represents the first letter of the German word Hoch (high), and the low tone corresponds to the letter T, which is the first letter of the German word Tief (low). In addition, the combinations of high tone—H and low tone—T were always mapped onto the same response fingers (e.g., index fingers or middle fingers of both hands). As a consequence, compatible responses required both index or both middle fingers. Incompatible responses were based on one index and one middle finger response. The difference between compatible and incompatible trials denotes the BCE. Stimuli of both tasks (S1 and S2) were presented with varying (40, 130, and 300 ms) stimulus onset asynchronies (SOA) to manipulate the temporal overlap between the two tasks, which was meant to emphasize task order and to reduce grouped responses.

Stimuli were presented in Arial font (font size 30) and in white on a black background with a viewing distance of approximately 60 cm. S1 was responded to with the left middle and index finger pressing the 'a' and 's' keys, respectively. Task 2 consisted of easy to discriminate auditory stimuli (S2), i.e., a high or low tone of 900 and 300 Hz, presented via a loudspeaker. Responses to S2 were provided by pressing the 'k' and 'l' keys using the right index and middle finger, respectively. Half of the participants responded to H and high tones with both index fingers and T and low tones with both middle fingers. The other half of the participants received the reversed assignment. Stimulus presentation and data recording were realized on a Pentium I computer with a Windows 10 platform using E-prime software (Version 3)^39^. Responses were collected on a German QWERTZ keyboard.

**Figure 1:**
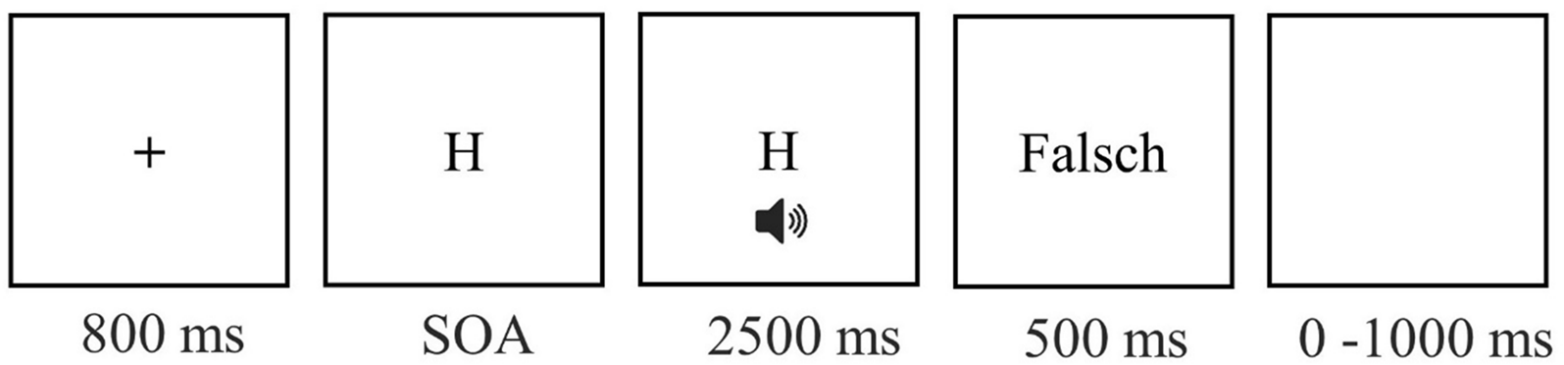
Trial procedure of the study. After the fixation sign, the visual stimulus for Task 1 (H or T) was presented. The auditory stimulus for Task 2 (high or low tone, represented by the speaker symbol) was delivered after one of three stimulus onset asynchronies (SOA, 40, 130, 300 ms). In the case of an erroneous response, feedback (i.e., “Falsch,” German for wrong) was presented.

The participants were instructed to respond first as fast and accurately as possible to S1 and only subsequently as fast and accurately as possible to S2. Each trial began with an 800 ms fixation period, in which a plus sign was presented in the center of the screen, followed by the onset of the visual S1. After a variable stimulus onset asynchrony (SOA) of 40, 130, or 300 ms, the auditory S2 was presented for 150 ms. S1 remained on screen for a maximum of 2500 ms (plus SOA duration) or until responses to both tasks were given. In the case of erroneous responses or wrong order, error feedback ("Falsch" or "Falsche Reihenfolge," respectively) was presented for 500 ms. The next trial started after a blank screen was presented for a random inter-trial-interval (ITI) between 0 and 1000 ms. Figure 1 depicts the trial procedure of the study.

In each session, the experiment started with 12 dual-task trials to instruct stimulus–response mappings, followed by one practice block and eight experimental blocks consisting of 72 trials each. The 72 trials consisted of 36 compatible and incompatible trials. Of the 36 trials, in each compatibility condition, the three SOAs were presented 12 times each. That is, each combination of 2(S1) × 2(S2) × 3(SOA) was presented six times per block. The practice block was excluded from the analysis. Thus, 576 trials per session were considered for analysis.

#### Stimulation protocol

**Figure 2:**
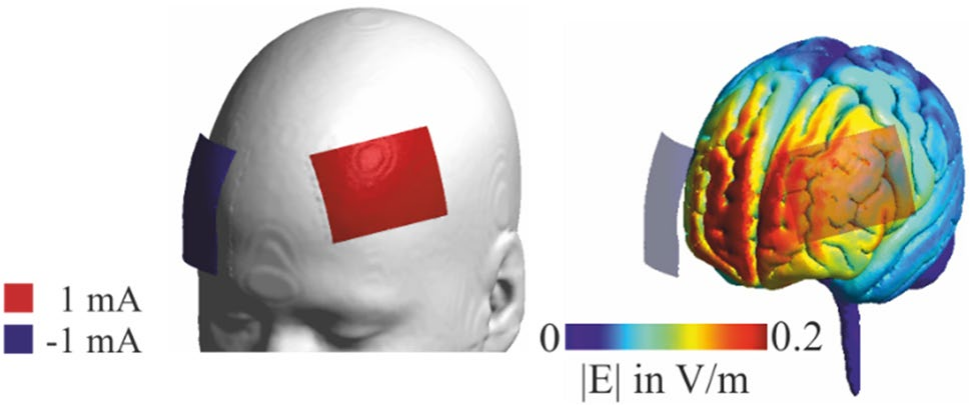
Demonstration of the electrode placement and simulation of electric field distribution of applied stimulation parameters on MNI head/brain with SimNibs (simnibs.org, Version 3)^40–42^. The stimulation electrode (red) was placed over F3, corresponding to the left dlPFC. The reference electrode (blue) was placed over Fp2, corresponding to the right supraorbital region.

Stimulation was delivered (NeuroConn DCStimulator Plus; neuroCare Grouo GmbH, Munich, Germany) using two saline-soaked surface sponge electrodes over the left dlPFC (anode, centered over F3, 10–20 EEG system, size: 5 × 7 cm^2^, current density = 0.028 mA/cm^2^ and the right supraorbital region (cathode, Fp2, 10–20 EEG system, size: 5 × 7 cm^2^, current density = 0.028 mA/cm^2^). Figure 2 (left) depicts the stimulation montage used, which is commonly applied in experiments targeting the dlPFC in multitasking^20,21^.

**Figure 3:**
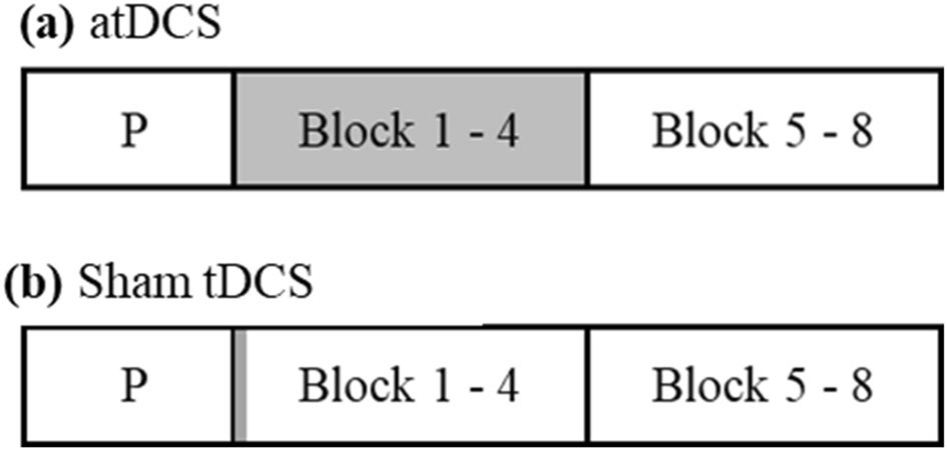
Experimental design. Illustration of atDCS and sham tDCS, which were realized in two sessions, separated approximately by 7 days. (**a**) For atDCS, stimulation began after the practice block and lasted 20 min through blocks 1 to 4 (shaded in grey). The second half of the experiment was not stimulated. (**b**) The stimulation lasted for 30 s in sham tDCS.

Both atDCS and sham tDCS started with a 10-s ramp on and ended with a 10-s ramp off. In atDCS, the stimulation started with the first experimental block and lasted until 20 min—through the first half of the experiment blocks, while the second half of the experiment was not stimulated (see Fig. 3). We simulated the electric field distribution of the applied stimulation parameters using computational modeling analyses on a standard brain (MNI) with SimNibs^40–42^, see Fig. 2 (right).

In the sham tDCS, the stimulation duration was 30 s, including 20 s of ramping^22,23^. The current intensity used for both stimulation conditions was 1.0 mA. The onset of stimulation produces a tingling or itching sensation in the beginning, thus effectively blinding them from the two conditions used^43^. At the end of the experiment, participants filled out a questionnaire to report the sensation of tDCS^44^ on a four-point scale (0 = none, 1 = mild, 2 = moderate, 3 = strong).

### Design

A 2 (Stimulation condition: atDCS, sham tDCS) × 2 (Compatibility: compatible, incompatible) × 3 (SOA: 40, 130, 300 ms) repeated measures single-blind, crossover, sham-controlled design was applied. Because BCEs take effect in Task 1 performance^45–47^, dependent measures were response times in Task 1 (RT1) and percent error in Task 1 (PE1). RT2 and PE2 are reported for completeness.

### Analysis

**Figure 4:**
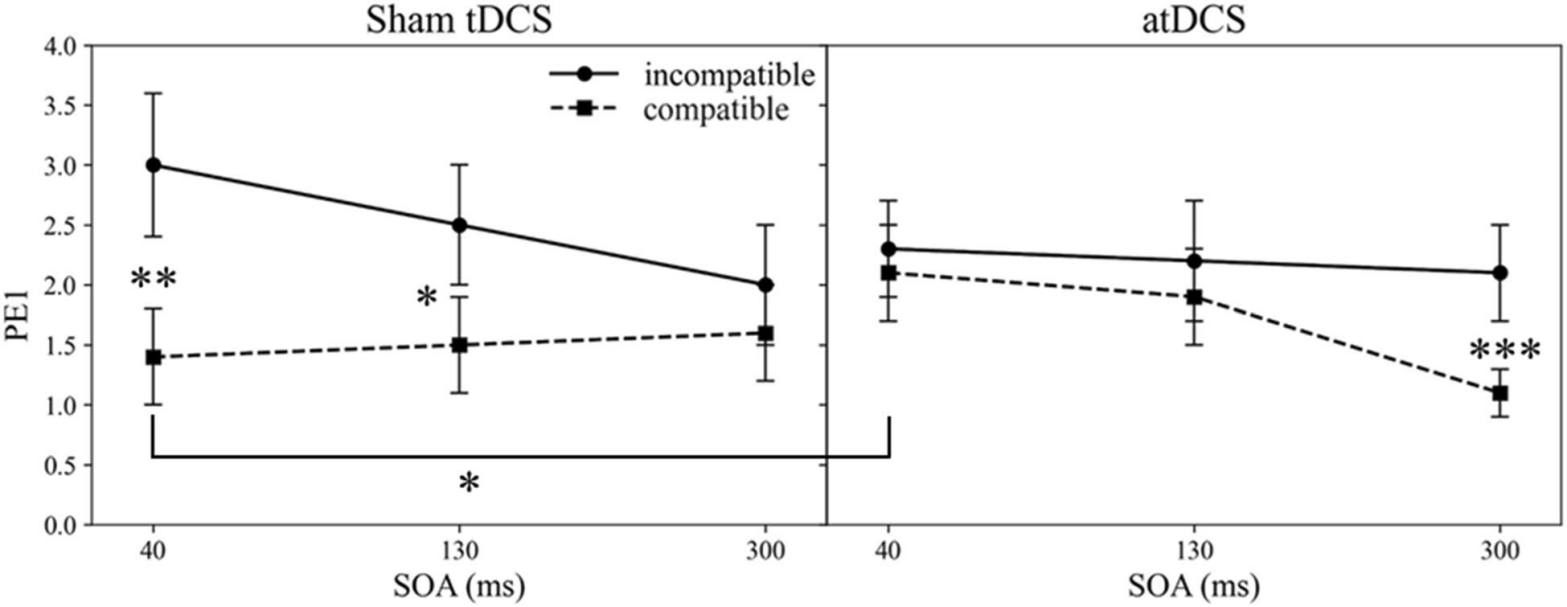
The percentage error in Task 1 (PE1) for compatible and incompatible trials across SOA (in ms), for atDCS and sham tDCS. Error bars represent standard error of the mean. * *p* ≤ 0.05, ** *p* ≤ 0.01, *** *p* ≤ 0.001.

The practice block of each session was not considered for analyses. Before RT analyses, erroneous trials in either Task 1 or Task 2 were removed. Further, all trials with RT1 < 200 ms and RT1 > 2000 ms and RT2 < 200 ms, and RT2 > 2500 ms were considered outliers and were not included in the analysis. This led to a removal of 0.6% of trials. A repeated measures ANOVA with the within-subject factors 2 (Stimulation condition: atDCS, sham tDCS) × 2 (Compatibility: compatible, incompatible) × 3 (SOA: 40, 130, 300 ms) was applied on mean RTs and PEs (see Tables 1, 2, 3 and Fig. 4). Greenhouse–Geisser corrections were applied when necessary.

**Table 1:** RT1 (in ms) as a function of Stimulation condition (atDCS vs. sham tDCS), SOA (40 vs. 130 vs. 300 ms), Compatibility (compatible vs. incompatible).

SOA	atDCS			sham tDCS		
	40	130	300	40	130	300
C	763 (29)	785 (31)	885 (31)	740 (29)	770 (29)	857 (32)
IC	841 (35)	866 (36)	947 (36)	821 (33)	852 (34)	908 (37)

Standard errors of the mean are presented in parentheses.

**Table 2:** PE2 as a function of Stimulation condition (atDCS vs. sham tDCS), SOA (40 vs. 130 vs. 300 ms), Compatibility (compatible vs. incompatible).

SOA	atDCS			sham tDCS		
	40	130	300	40	130	300
C	2.5 (0.6)	2.7 (0.7)	2.8 (0.6)	2.3 (0.6)	2.0 (0.5)	2.5 (0.5)
IC	5.0 (0.8)	3.7 (0.7)	3.4 (0.6)	4.4 (0.8)	3.9 (0.9)	2.6 (0.5)

Standard errors of the mean are presented in parentheses.

**Table 3:** RT2 (in ms) as a function of Stimulation condition (atDCS vs. sham tDCS), SOA (40 vs. 130 vs. 300 ms), Compatibility (compatible vs. incompatible).

SOA	atDCS			sham tDCS		
	40	130	300	40	130	300
C	908 (32)	844 (33)	768 (32)	894 (31)	841 (31)	751 (30)
IC	997 (36)	939 (37)	840 (36)	992 (34)	935 (34)	815 (35)

Standard errors of the mean are presented in parentheses.

## Results

### PE1

Participants made more errors in Task 1 when the Task 2 response was incompatible (*M* = 2.4%, *SE* = 0.4) as compared to compatible to the Task 1 response (*M* = 1.6%, *SE* = 0.3), *F*(1, 33) = 10.29, *p* = 0.003, η_p_^2^ = 0.24, representing the existence of a BCE. The factor SOA, representing the temporal overlap between the two tasks, was statistically not significant, *F*(2, 66) = 2.83, *p* = 0.066, η_p_^2^ = 0.08. Overall, the error rates did not differ between Stimulation condition, *F* < 1.

Importantly, the application of atDCS modulated the BCE as compared to sham tDCS across the three SOAs, as indicated by the significant three-way interaction between Stimulation condition, SOA, and Compatibility, *F*(2, 66) = 5.50, *p* = 0.006, *η*_*p*_^2^ = 0.14. As illustrated in Fig. 4, the impact of atDCS on task shielding was largest at conditions of high temporal task overlap, as the BCE was eliminated at the shorter SOAs, *t*s < 1, but present at the longest SOA, *t*(33) = − 3.58, *p* = 0.001. Sham tDCS, however, showed the typical pattern of decreasing BCEs with increasing SOAs (e.g.,^45,48^), with significant BCEs at the two shortest SOAs, *t*(33) = − 2.94, *p* = 0.006 (SOA 40 ms) and *t*(33) = − 2.21, *p* = 0.034 (SOA 130 ms), but not at the longest SOA of 300 ms, *t*(33) = − 1.38, *p* = 0.176 (see Fig. 3). Not surprisingly, the most pronounced reduction of the BCE by atDCS compared to sham tDCS was obtained at the shortest SOA of 40 ms, *F*(1, 33) = 5.96, *p* = 0.020, η_p_^2^ = 0.15. The difference in BCE between atDCS and sham tDCS was not significant at SOA 130 ms, *F*(1, 33) = 1.13, *p* = 0.295, η_p_^2^ = 0.03, and slightly reversed for the longest SOA of 300 ms, *F*(1, 33) = 6.01, *p* = 0.020, η_p_^2^ = 0.15. This SOA-dependent pattern of the impact of stimulation on the BCE explains the overall lack of the interaction between Stimulation condition and Compatibility, *F*(1, 33) = 1.57, *p* = 0.219, η_p_^2^ = 0.05. No further effects were significant, *Fs* < 1.57, *ps* > 0.219.

To test for potential effects of stimulation condition order (i.e., atDCS first vs. sham first), an analysis with stimulation order as an additional between-subject factor was conducted. However, no significant main effect or interaction with stimulation order was observed, all *p*’s > 0.066. Finally, an additional factor Stimulation Block (online stimulation vs. offline stimulation), did not further modulate the interaction between Stimulation Condition x SOA x Compatibility, *F* < 1, suggesting that the effects of atDCS were not restricted to the online stimulation period but extended into the offline stimulation blocks as well.

### RT1

Participants responded faster in compatible trials (*M* = 800 ms, *SE* = 29) as compared to incompatible trials (*M* = 872 ms, *SE* = 33), confirming a BCE, *F*(1, 33) = 34.85, *p* < 0.001, η_p_^2^ = 0.51.

There was also a significant main effect of SOA, *F*(1.20, 39.45) = 163.40, *p* < 0.001, η_p_^2^ = 0.83, with increasing RT1 the larger the SOA (i.e., *M* = 791 ms, *SE* = 30; *M* = 818 ms, *SE* = 31 and *M* = 899 ms, *SE* = 32 for SOAs 40, 130 and 300 ms, respectively). This increase was slightly more pronounced for atDCS than for sham tDCS, as revealed by the significant interaction between Stimulation condition and SOA, *F*(2, 66) = 3.72, *p* = 0.030, η_p_^2^ = 0.10. However, differences between atDCS and sham tDCS at each SOA condition remained descriptively, as they did not exceed the statistical significance level (all *p*'s > 0.09). Furthermore, RT1s did not generally differ between Stimulation conditions, *F*(1, 33) = 1.83, *p* = 0.185, η_p_^2^ = 0.05. Finally, the BCE was modulated by SOA, *F*(2, 66) = 9.12, *p* < 0.001, η_p_^2^ = 0.22, with the largest BCEs for the two shortest SOAs (*M* = 79 ms, *SE* = 43 and *M* = 82 ms, *SE* = 44 for SOAs 40 and 130, respectively) and a somewhat smaller BCE for the SOA 300 ms (*M* = 57 ms, *SE* = 46). However, the three-way interaction between Stimulation condition, SOA, and Compatibility, and other interactions were not significant, *Fs* < 1.

### PE2

Mean PE2 is summarized in Table 2. The main effect of Compatibility, *F*(1, 33) = 22.28, *p* < 0.001, η_p_^2^ = 0.40, was significant. Participants made more errors in incompatible (*M* = 3.8%, *SE* = 0.6) as compared to compatible trials (*M* = 2.5%, *SE* = 0.5). The factor SOA did not reach statistical significance, *F*(1.56, 51.52) = 3.19, *p* = 0.061, η_p_^2^ = 0.09. The SOA x Compatibility interaction was significant, *F*(1.67, 55.10) = 8.62, *p* = 0.001, η_p_^2^ = 0.21, with the largest forward-crosstalk at 40 ms (*M* = 2.3%, *SE* = 0.9), followed by 130 ms (*M* = 1.5%, *SE* = 0.9) and the lowest at 300 ms (*M* = 0.4%, *SE* = 0.7). No further effects were significant, *Fs* < 1.50, *ps* > 1.0.

### RT2

Mean correct RT2 is summarized in Table 3. Participants were faster in compatible (*M* = 834 ms, *SE* = 30) than incompatible (*M* = 920 ms, *SE* = 33) trials, *F*(1, 33) = 40.29, *p* < 0.001, η_p_^2^ = 0.55. The main effect of SOA was significant, *F*(1.32, 43.67) = 370.02, *p* < 0.001, η_p_^2^ = 0.92, confirming a decrease in RT2 as the SOA increases (i.e., *M* = 948 ms, *SE* = 31; *M* = 890 ms, *SE* = 31, and *M* = 794 ms, *SE* = 31 for SOAs 40, 130 and 300 ms, respectively). The decrease was slightly more pronounced for sham tDCS (*M* = 943 ms, *SE* = 32; *M* = 888 ms, *SE* = 31 and *M* = 783 ms, *SE* = 32 for SOA 40, 130 and 300, respectively) than atDCS (*M* = 952 ms, *SE* = 34; *M* = 891 ms, *SE* = 35 and *M* = 804 ms, *SE* = 33 for SOA 40, 130 and 300, respectively), as indicated by the significant Stimulation condition x SOA interaction, *F*(2, 66) = 3.58, *p* = 0.033, η_p_^2^ = 0.10. However, RT2 did not differ between sham and atDCS condition at either SOA (all *p*’s > 0.338). The Compatibility x SOA interaction was significant, *F*(2, 66) = 10.15, *p* < 0.001, η_p_^2^ = 0.24, with the largest forward crosstalk for the two shortest SOAs (*M* = 94 ms, *SE* = 45 ms and *M* = 95 ms*, SE* = 45 ms for SOAs 40 and 130, respectively), and a smaller forward crosstalk of 68 ms (*SE* = 44) when the SOA was 300 ms.

### General tDCS effects

The frequency and severity of reported side effects (i.e., itching, pain, burning, heat, metallic taste, and fatigue) were assessed for the last experimental session. None of the participants reported metallic taste as a side effect in atDCS or sham tDCS. Two-sided Fisher exact tests revealed no association between the frequency of reported side effects and type of stimulation (atDCS and sham tDCS), all *p*’s > 0.053. Severity ratings were compared using Mann–Whitney-U-Test with exact significance values due to the small sample size (Table 4). Furthermore, at the end of the experiment, participants were asked to identify or guess which type of stimulation was administered in each of the two sessions: 47.1% guessed correctly, 23.5% guessed incorrectly, and 29.4% had no guess. A Chi-squared test revealed no difference in the observed proportion amongst the different response options, *X*^*2*^(2, 34) = 3.06, *p* = 0.217. These guessing rates are similar to other studies that report values of comparable magnitude using a crossover, double-blind design^49^.

**Table 4:** Severity ratings of experienced side effects after the last experimental session.

Side effects	atDCS median (range)	sham tDCS median (range)	*p* value (Mann–Whitney-U-Test)
Itching	1 (0–3)	1 (0–3)	0.218
Pain	0 (0–2)	0 (0–2)	0.114
Burning	1 (0–3)	0 (0–2)	0.067
Heat	0 (0–2)	0 (0–1)	0.946
Fatigue	1 (0–3)	2 (0–3)	0.306

## Discussion

The present study investigated whether modulation of excitability in the left-lateralized executive control network, as modulated by a prefrontal tDCS setup with anode over left dlPFC, facilitated the executive control function of prioritized task shielding in dual tasking. Results demonstrated that atDCS, as compared to sham tDCS, reduced crosstalk between the two tasks indicating improved task shielding. Importantly, improved task shielding was observed in conditions of highest dual-task demands, i.e., the largest temporal overlap between the two tasks.

More precisely, in the condition of sham tDCS, crosstalk was stronger the more simultaneously both tasks were being processed. The close temporal proximity of stimuli at high temporal task overlap allows for more simultaneous task-component processing, which typically increases crosstalk between tasks. With less temporal task overlap, crosstalk typically declines. Thus, our findings in the sham tDCS condition mirror the usual result pattern obtained in dual-task research without any brain stimulation (e.g.,^45,48,50^). In contrast, the administration of atDCS over left dlPFC completely eliminated the backward crosstalk effect, especially in conditions of highest interference vulnerability, i.e., when both tasks were performed virtually at the same time (short SOA).

The findings of the present interventional approach, therefore, extend previous imaging studies suggesting a significant role of the dlPFC in task shielding (e.g.,^16^). We could show that modulation of the excitability in the left-lateralized executive control network can improve the cognitive control function of task shielding and, thus, interference control in dual-task processing. The fact that the effect of atDCS is especially pronounced in conditions of highest interference vulnerability is in line with previous behavioral studies demonstrating adaptively increased task shielding when high-interference situations are predicted, such as high frequency of short SOAs^34^ or high probability of between-task conflict^51,52^.

Based on the present observations, we can only speculate how the modulation of the underlying executive control network by atDCS over left dlPFC facilitates processes of task shielding. One way of interpreting our results would be that atDCS could have aided conflict resolution by biasing the signals needed for Task 1 prioritized processing. Recent neuroimaging studies, for example, have shown a conflict-related attentional biasing in favor of task-relevant stimulus dimensions in single tasks^53–56^.

Alternatively, tDCS-induced facilitation of task shielding may be directly related to enhanced processing efficiency rather than conflict resolution. That is, neural accounts to explain training effects on cognitive task performance postulate that training can lead to more efficient processing of the already engaged neural circuits^57,58^. Similarly, atDCS could have increased the excitability of left dlPFC, creating a more efficient functional adaptation and processing specifically within the dlPFC (e.g.,^6^). As a consequence, stimulus–response translations for each task would run more efficiently under atDCS, giving less opportunity for crosstalk.

Such an assumed benefit in neural efficiency, however, did not show up in the present RT measures, as the effects of improved task shielding under atDCS were found in error rates only. A selective impact of tDCS on error rates is not uncommon and has been reported frequently^23,26,59^. Frings et al.^59^, for example, speculated that "better” or “worse” functioning of the dlPFC would not necessarily translate in modulations of overall RTs, but could alter the ratio of trials in which its function of interference control works effectively or fails.

In contrast to the error rates, RT results showed an increase of RT1 with longer SOAs. In dual-task paradigms with mostly short SOAs, participants tend to execute both responses at the same time, which leads to a typical strategic Task 1 response deferment with increasing SOA^60,61^. This strategic response deferment was slightly more pronounced for atDCS compared to sham stimulation and also accounts for the weaker decline of RT2 across SOA for atDCS compared to sham stimulation—the more delayed the Task 1 response at SOA 300, the later the response of Task 2. Most importantly, however, this RT pattern does not compromise the findings and their interpretation of atDCS modulating the size of the BCE and the extent of task shielding in Task 1.

While the present results demonstrate that atDCS over the dlPFC improves task shielding in conditions of high between-task interference, further research combining functional neuroimaging and brain stimulation techniques may be informative to which extent facilitated task shielding is a consequence of tDCS-modulated activity and/or functional connectivity in underlying brain networks. In addition, the present effects of tDCS on task shielding were assessed across all experimental blocks, i.e., during and immediately after stimulation^20,22^. Further studies should test the duration of tDCS-induced improved task shielding, as previous studies reported that anodal tDCS over left dlPFC can reduce performance costs in dual-task compared to single-task performance up to an hour after stimulation^21^. Finally, the present study investigated only young adults. Since age-related differences in task shielding have recently been reported^62,63^, it remains unclear if other age groups would benefit in the same way from stimulation.

## Limitations

The present study used a conventional tDCS setup as applied in previous studies that reported successful tDCS-related dual-task performance benefits^21–23^. We are aware that tDCS with this setup over left dlPFC allows for modulation of the activity and/or connectivity in the underlying executive control network. In order to test for a causal role of the involvement of the dlPFC in increased task shielding, targeted stimulation of focal brain regions, e.g., by high-definition (HD) tDCS (e.g.,^49,64,65^) would be is required. The use of an active control group allows checking for specificity of tDCS effects^66^ and to avoid blinding issues arising from perceptual sensation^67^. It becomes even more important if inefficient blinding leads to increase in demand characteristics, such as arousal or motivation on performance^68^. Because potential sensory co-stimulation confounds cannot be ruled out with certainty, the use of an active control group, carefully matched for sensation and irritation, is becoming an important necessity and should be considered in future studies^69^.

In addition, to potentially increase blinding efficacy, a double-blind design might be taken into consideration. We carefully implemented certain protocols to standardize testing across all participants, such as written instructions, and minimized interactions with participants^70^ to prevent an experimenter bias. However, to safely exclude the possibility of even unconscious experimenter biases, future research should consider double-blind designs. Because the effects of atDCS on task shielding were obtained in errors, subsequent studies using similar paradigms with typically low error rates^22,23^ should ensure a sufficiently high number of errors in the experiment.

## Summary

We investigated the effects of atDCS over left dlPFC on shielding of prioritized task processing in dual tasks. We found that atDCS reduced between-task interference, especially at high temporal overlap between tasks as compared to sham tDCS. These findings extend previous behavioral and neuroimaging studies by demonstrating that stimulating the left-lateralized executive control network can improve dual performance by reducing processing interactions between simultaneously performed tasks. This atDCS-induced improvement of cognitive function was most evident in conditions of highest vulnerability to between-task interference. Given the increasing prevalence of multitasking in modern life, this suggests that atDCS should be explored as an intervention tool in future studies, for example, with repeated training and application of atDCS in individuals in advanced age that are known to be susceptible to impairments in multitasking performance.

## Data Availability

The datasets generated during the current study are available in the PsychArchives repository, 10.23668/psycharchives.8155.
